# Analysis of the Influence of microRNAs in Lithium Response in Bipolar Disorder

**DOI:** 10.3389/fpsyt.2018.00207

**Published:** 2018-05-31

**Authors:** Céline S. Reinbold, Andreas J. Forstner, Julian Hecker, Janice M. Fullerton, Per Hoffmann, Liping Hou, Urs Heilbronner, Franziska Degenhardt, Mazda Adli, Kazufumi Akiyama, Nirmala Akula, Raffaella Ardau, Bárbara Arias, Lena Backlund, Antonio Benabarre, Susanne Bengesser, Abesh K. Bhattacharjee, Joanna M. Biernacka, Armin Birner, Cynthia Marie-Claire, Pablo Cervantes, Guo-Bo Chen, Hsi-Chung Chen, Caterina Chillotti, Scott R. Clark, Francesc Colom, David A. Cousins, Cristiana Cruceanu, Piotr M. Czerski, Alexandre Dayer, Bruno Étain, Peter Falkai, Louise Frisén, Sébastien Gard, Julie S. Garnham, Fernando S. Goes, Paul Grof, Oliver Gruber, Ryota Hashimoto, Joanna Hauser, Stefan Herms, Stéphane Jamain, Esther Jiménez, Jean-Pierre Kahn, Layla Kassem, Sarah Kittel-Schneider, Sebastian Kliwicki, Barbara König, Ichiro Kusumi, Nina Lackner, Gonzalo Laje, Mikael Landén, Catharina Lavebratt, Marion Leboyer, Susan G. Leckband, Carlos A. López Jaramillo, Glenda MacQueen, Mirko Manchia, Lina Martinsson, Manuel Mattheisen, Michael J. McCarthy, Susan L. McElroy, Marina Mitjans, Francis M. Mondimore, Palmiero Monteleone, Caroline M. Nievergelt, Urban Ösby, Norio Ozaki, Roy H. Perlis, Andrea Pfennig, Daniela Reich-Erkelenz, Guy A. Rouleau, Peter R. Schofield, K. Oliver Schubert, Barbara W. Schweizer, Florian Seemüller, Giovanni Severino, Tatyana Shekhtman, Paul D. Shilling, Kazutaka Shimoda, Christian Simhandl, Claire M. Slaney, Jordan W. Smoller, Alessio Squassina, Thomas J. Stamm, Pavla Stopkova, Sarah K. Tighe, Alfonso Tortorella, Gustavo Turecki, Julia Volkert, Stephanie H. Witt, Adam J. Wright, L. Trevor Young, Peter P. Zandi, James B. Potash, J. Raymond DePaulo, Michael Bauer, Eva Reininghaus, Tomáš Novák, Jean-Michel Aubry, Mario Maj, Bernhard T. Baune, Philip B. Mitchell, Eduard Vieta, Mark A. Frye, Janusz K. Rybakowski, Po-Hsiu Kuo, Tadafumi Kato, Maria Grigoroiu-Serbanescu, Andreas Reif, Maria Del Zompo, Frank Bellivier, Martin Schalling, Naomi R. Wray, John R. Kelsoe, Martin Alda, Francis J. McMahon, Thomas G. Schulze, Marcella Rietschel, Markus M. Nöthen, Sven Cichon

**Affiliations:** ^1^Human Genomics Research Group, Department of Biomedicine, University of Basel, Basel, Switzerland; ^2^Institute of Medical Genetics and Pathology, University Hospital Basel, Basel, Switzerland; ^3^Institute of Human Genetics, University of Bonn, Bonn, Germany; ^4^Department of Genomics, Life & Brain Center, University of Bonn, Bonn, Germany; ^5^Department of Psychiatry (UPK), University of Basel, Basel, Switzerland; ^6^Department of Biostatistics, Harvard T.H. Chan School of Public Health, Boston, MA, United States; ^7^Neuroscience Research Australia, Sydney, NSW, Australia; ^8^School of Medical Sciences, University of New South Wales, Sydney, NSW, Australia; ^9^Intramural Research Program, US Department of Health & Human Services, National Institute of Mental Health, National Institutes of Health, Bethesda, MD, United States; ^10^Department Psychiatry and Psychotherapy, Institute of Psychiatric Phenomics and Genomics, University Hospital, LMU Munich, Munich, Germany; ^11^Department of Psychiatry and Psychotherapy, Charité Universitätsmedizin, Berlin, Germany; ^12^Department of Biological Psychiatry and Neuroscience, Dokkyo Medical University School of Medicine, Mibu, Japan; ^13^Unit of Clinical Pharmacology, University Hospital of Cagliari, Cagliari, Italy; ^14^Zoology and Biological Anthropology Section (Department of Evolutive Biology, Ecology and Environmental Sciences), Facultat de Biologia and Institut de Biomedicina, CIBERSAM, Universitat de Barcelona, Barcelona, Spain; ^15^Department of Molecular Medicine and Surgery, Karolinska Institutet and Center for Molecular Medicine, Karolinska University Hospital, Stockholm, Sweden; ^16^Bipolar Disorder Program, Institute of Neuroscience, Hospital Clinic, CIBERSAM, IDIBAPS, University of Barcelona, Barcelona, Spain; ^17^Special Outpatient Center for Bipolar Affective Disorder, Medical University of Graz, Graz, Austria; ^18^Health Sciences Research, Mayo Clinic, Rochester, MN, United States; ^19^Department of Psychiatry and Psychology, Mayo Clinic, Rochester, MN, United States; ^20^Institut National de la Santé et de la Recherche Médicale, U1144, Paris, France; ^21^Université Paris Diderot, Sorbonne Paris Cité, UMR-S 1144, Paris, France; ^22^Department of Psychiatry, University of California, San Diego, San Diego, CA, United States; ^23^The Neuromodulation Unit, McGill University Health Centre, Montreal, QC, Canada; ^24^Queensland Brain Institute, The University of Queensland, Brisbane, QLD, Australia; ^25^Department of Psychiatry & Center of Sleep Disorders, National Taiwan University Hospital, Taipei, Taiwan; ^26^Discipline of Psychiatry, University of Adelaide, Adelaide, SA, Australia; ^27^Mental Health Research Group, IMIM-Hospital del Mar, Barcelona, Spain; ^28^Campus for Ageing and Vitality, Newcastle University, Newcastle upon Tyne, United Kingdom; ^29^Douglas Mental Health University Institute, McGill University, Montreal, QC, Canada; ^30^Psychiatric Genetic Unit, Poznan University of Medical Sciences, Poznan, Poland; ^31^Mood Disorders Unit, HUG - Geneva University Hospitals, Geneva, Switzerland; ^32^AP-HP, GH Saint-Louis – Lariboisière – F. Widal, Département de Psychiatrie et de Médecine Addictologique, Paris, France; ^33^Department of Psychiatry and Psychotherapy, Ludwig-Maximilians-University Munich, Munich, Germany; ^34^Service de Psychiatrie, Hôpital Charles Perrens, Bordeaux, France; ^35^Department of Psychiatry, Dalhousie University, Halifax, NS, Canada; ^36^Department of Psychiatry and Behavioral Sciences, Johns Hopkins University, Baltimore, MD, United States; ^37^Mood Disorders Center of Ottawa, Ottawa, ON, Canada; ^38^Department of Psychiatry and Psychotherapy, University Medical Center, Georg-August University Göttingen, Göttingen, Germany; ^39^Molecular Research Center for Children's Mental Development, United Graduate School of Child Development, Osaka University, Osaka, Japan; ^40^Institut National de la Santé et de la Recherche Médicale U955, Psychiatrie Translationnelle, Créteil, France; ^41^Service de Psychiatrie et Psychologie Clinique, Centre Psychothérapique de Nancy - Université de Lorraine, Nancy, France; ^42^Department of Psychiatry, Psychosomatic Medicine and Psychotherapy, University Hospital Frankfurt, Frankfurt, Germany; ^43^Department of Adult Psychiatry, Poznan University of Medical Sciences, Poznan, Poland; ^44^Department of Psychiatry and Psychotherapeuthic Medicine, Landesklinikum Neunkirchen, Neunkirchen, Austria; ^45^Department of Psychiatry, Hokkaido University Graduate School of Medicine, Sapporo, Japan; ^46^Institute of Neuroscience and Physiology, The Sahlgrenska Academy at the Gothenburg University, Gothenburg, Sweden; ^47^Department of Medical Epidemiology and Biostatistics, Karolinska Institutet, Stockholm, Sweden; ^48^Assistance Publique-Hôpitaux de Paris, Hôpital Albert Chenevier - Henri Mondor, Pôle de Psychiatrie, Créteil, France; ^49^Department of Pharmacy, VA San Diego Healthcare System, San Diego, CA, United States; ^50^Department of Psychiatry, University of Antioquia, Medellín, Medellín, Colombia; ^51^Department of Psychiatry, University of Calgary, Calgary, AB, Canada; ^52^Section of Psychiatry, Department of Medical Sciences and Public Health, University of Cagliari, Cagliari, Italy; ^53^Department of Pharmacology, Dalhousie University, Halifax, NS, Canada; ^54^Department of Clinical Neurosciences, Karolinska Institutet, Stockholm, Sweden; ^55^Department of Biomedicine, Aarhus University, Aarhus, Denmark; ^56^Department of Psychiatry, VA San Diego Healthcare System, San Diego, CA, United States; ^57^Department of Psychiatry, Lindner Center of Hope/University of Cincinnati, Mason, OH, United States; ^58^Neurosciences Section, Department of Medicine and Surgery, University of Salerno, Salerno, Italy; ^59^Department of Psychiatry, University of Campania “Luigi Vanvitelli”, Naples, Italy; ^60^Department of Neurobiology, Care Sciences, and Society, Karolinska Institutet and Center for Molecular Medicine, Karolinska University Hospital, Stockholm, Sweden; ^61^Department of Psychiatry, Nagoya University Graduate School of Medicine, Nagoya, Japan; ^62^Department of Psychiatry, Massachusetts General Hospital and Harvard Medical School, Boston, MA, United States; ^63^Department of Psychiatry and Psychotherapy, Medical Faculty, University Hospital Carl Gustav Carus, Technische Universität Dresden, Dresden, Germany; ^64^Department of Neurology and Neurosurgery, Montreal Neurological Institute and Hospital, McGill University, Montreal, QC, Canada; ^65^Mental Illness, Neuroscience Research Australia, Sydney, NSW, Australia; ^66^Department of Biomedical Sciences, University of Cagliari, Cagliari, Italy; ^67^Department of Psychiatry, Dokkyo Medical University School of Medicine, Mibu, Japan; ^68^Medical school, Sigmund Freud University, Vienna, Austria; ^69^Bipolar Center Wiener Neustadt, Vienna, Austria; ^70^Department of Psychiatry, Psychotherapy and Psychosomatics, Medical School Brandenburg, Neuruppin, Germany; ^71^Department of Psychiatry, National Institute of Mental Health, Klecany, Czechia; ^72^Department of Psychiatry, University of Iowa, Iowa, IA, United States; ^73^Department of Genetic Epidemiology in Psychiatry, Medical Faculty Mannheim, Central Institute of Mental Health, University of Heidelberg, Mannheim, Germany; ^74^School of Psychiatry, University of New South Wales, and Black Dog Institute, Sydney, NSW, Australia; ^75^Department of Psychiatry, University of British Columbia, Vancouver, BC, Canada; ^76^Department of Mental Health, Johns Hopkins Bloomberg School of Public Health, Baltimore, MD, United States; ^77^Department of Public Health and Institute of Epidemiology and Preventive Medicine, National Taiwan University, Taipei, Taiwan; ^78^Laboratory for Molecular Dynamics of Mental Disorders, RIKEN Brain Science Institute, Saitama, Japan; ^79^Biometric Psychiatric Genetics Research Unit, Alexandru Obregia Clinical Psychiatric Hospital, Bucharest, Romania; ^80^Research Centre Jülich, Institute of Neuroscience and Medicine (INM-1), Jülich, Germany

**Keywords:** bipolar disorder, lithium response, microRNA, common variants, genome-wide association study

## Abstract

Bipolar disorder (BD) is a common, highly heritable neuropsychiatric disease characterized by recurrent episodes of mania and depression. Lithium is the best-established long-term treatment for BD, even though individual response is highly variable. Evidence suggests that some of this variability has a genetic basis. This is supported by the largest genome-wide association study (GWAS) of lithium response to date conducted by the International Consortium on Lithium Genetics (ConLiGen). Recently, we performed the first genome-wide analysis of the involvement of miRNAs in BD and identified nine BD-associated miRNAs. However, it is unknown whether these miRNAs are also associated with lithium response in BD. In the present study, we therefore tested whether common variants at these nine candidate miRNAs contribute to the variance in lithium response in BD. Furthermore, we systematically analyzed whether any other miRNA in the genome is implicated in the response to lithium. For this purpose, we performed gene-based tests for all known miRNA coding genes in the ConLiGen GWAS dataset (*n* = 2,563 patients) using a set-based testing approach adapted from the versatile gene-based test for GWAS (VEGAS2). In the candidate approach, *miR-499a* showed a nominally significant association with lithium response, providing some evidence for involvement in both development and treatment of BD. In the genome-wide miRNA analysis, 71 miRNAs showed nominally significant associations with the dichotomous phenotype and 106 with the continuous trait for treatment response. A total of 15 miRNAs revealed nominal significance in both phenotypes with *miR-633* showing the strongest association with the continuous trait (*p* = 9.80E-04) and *miR-607* with the dichotomous phenotype (*p* = 5.79E-04). No association between miRNAs and treatment response to lithium in BD in either of the tested conditions withstood multiple testing correction. Given the limited power of our study, the investigation of miRNAs in larger GWAS samples of BD and lithium response is warranted.

## Introduction

Bipolar disorder (BD) is a severe neuropsychiatric condition categorized by recurrent episodes of depression and mania. BD is common, with a lifetime prevalence of around 1% in the general population (1). The elevated morbidity and mortality, the typically early age at onset in young adulthood and the chronic course of BD make it a major public health problem, and BD is classified as one of the top 25 leading causes of the global burden of disease (2). Epidemiological and molecular genetic data strongly suggest that BD is a complex disorder (3) which means that both genetic and environmental factors influence illness risk. Based on twin studies the overall heritability of BD has been estimated to be over 70% (4, 5), suggesting a substantial involvement of genetic factors in the development of the disease.

Mood stabilizers are used as the first-line mode of medication in the treatment of BD (6). Amongst these drugs, lithium is used as a preventive agent for manic and depressive episodes (7), suicide attempts, and death by suicide, and shows the greatest support for long-term relapse prevention (8, 9). Consequently, lithium is endorsed as a first-line and best-established long-term treatment for BD, even though individual response is highly variable (6, 8, 10). Evidence suggests that some of the variability in lithium response has a genetic basis (11, 12). This hypothesis is supported by the largest genome-wide association study (GWAS) of lithium response to date, which was conducted by the International Consortium on Lithium Genetics (ConLiGen) (13, 14). The study investigated genomic data of 2,563 BD patients, identifying a genome-wide significant locus on chromosome 21, which contains two long, non-coding RNA genes (lncRNAs) (14).

Non-coding RNAs (ncRNAs) are transcribed from DNA but do not encode protein, and are involved in complex mechanisms of gene regulation, particularly in fine regulation of the timing and level of expression of their target genes. Another class of ncRNAs whose role in the pathophysiology of psychiatric disorders is emerging, is that of microRNAs (miRNAs). miRNAs are short RNA molecules, which in the mature processed form are 21 to 25-nucleotides in length, that work as post-transcriptional regulators of gene expression (15). To create a mature miRNA, a primary miRNA (pri-miRNA, typically >1,000 nucleotides in length) is first transcribed, and forms a secondary structure through self-base pairing (16, 17). This is cleaved by the Drosha-DiGeorge syndrome critical region gene 8 (Drosha-DGCR8) complex to create a pre-miRNA of around 70 nucleotides (16). This double stranded RNA is exported from the nucleus, cleaved by Dicer-transactivation-responsive RNA-binding protein (Dicer-TRBP) to form the mature miRNA (16), which can then target complementary messenger RNA (mRNA) transcripts through the RNA-induced silencing complex (RISC) to regulate expression (e.g., via mRNA degradation or translational repression) (18). Several studies have reported that miRNAs are potential predictors of treatment response in complex genetic disorders (19–21) including lithium response in BD (22). Furthermore, miRNAs are implicated in biological pathways that regulate brain development and synaptic plasticity (23, 24). Indeed, *miR-137* has emerged as a key risk gene in schizophrenia, and is known to regulate the expression of several genes that are independently associated with schizophrenia (25, 26). This implies the potential involvement of miRNAs in the pathogenesis of psychiatric disorders including BD. This hypothesis is further supported by the results of a large GWAS of BD (27) where a single-nucleotide polymorphism (SNP) flanking *miR-2113* was amongst the strongest findings.

Our group performed the first genome-wide analysis of the involvement of miRNAs in the development of BD, in a sample of 9,747 patients and 14,278 controls (28) in which we identified nine BD-associated miRNAs that withstood stringent Bonferroni-correction for multiple testing. However, it is largely unknown whether these miRNAs are also associated with lithium response in BD.

Therefore, the aim of the present study was to determine whether common variants at any of the nine BD-associated miRNAs contribute to the variance in lithium response in BD. Furthermore, we systematically analyzed whether any other miRNA is implicated in the response to lithium. For this purpose, we performed window-based association testing for all known miRNA coding genes in the largest GWAS dataset of lithium response so far.

## Materials and methods

### Sample description

Analyses were performed using summary statistics from the previously published GWAS of lithium response in BD patients (*n* = 2,563 patients) (14). These GWAS datasets were collected by ConLiGen and combine imputed genotype data from 22 contributing sites from four continents (Europe, America, Asia, and Australia). The study was approved by the respective local ethics committees. Written informed consent was obtained from all participants prior to inclusion. The Alda scale was used to create a dichotomous (good vs. poor response to lithium) and a continuous measure (range 0–10) for the evaluation of long-term treatment response to lithium. Briefly, the Alda scale measures symptom improvement in the course of lithium treatment (A score, range 0–10), which is then weighted against five criteria (B score) that assess confounding factors, each scored 0, 1, or 2. The total score is calculated by subtracting the total B score from the A score. Negative scores are set to 0 by default leading to a total score range from 0 to 10. For the purpose of the present analysis, subjects with a total score of 7 or higher were defined as showing “good response” to lithium treatment in the dichotomous phenotype. As continuous measurement, we used the A score, but excluded all individuals with a total B score greater than 4, as continuous measure (14, 29).

### Definition of candidate and genome-wide miRNAs

Information on the nine BD-associated miRNAs was obtained from our previously published genome-wide analysis of miRNAs in BD (28). The chromosomal positions of the miRNAs were obtained from the miRBase database (release 21) (30).

For the genome-wide miRNA association analysis chromosomal positions for all 1,871 remaining miRNAs were obtained from miRBase (release 21). miRNAs which were not located on autosomal chromosomes (*n* = 120) were removed from further analysis. Only miRNA genes which were covered in the summary statistics of lithium response were included, resulting in 1,692 miRNAs which were tested in the genome-wide analysis. For each gene, the entire preprocessed transcript ± 20 kilobase (kb) flanking sequence were analyzed, which would include the majority of the regulatory regions (17).

### MiRNA-based association tests

For the gene-based tests, we applied a set-based testing approach adapted from the versatile gene-based test for GWAS (VEGAS2) (31, 32) with a minor correction for the top-0.1-test option (33). This algorithm is obtainable upon request. The top-0.1-test was used since it showed the highest sensitivity with less than 1% false positives across a variety of investigated gene-level methods (34). The applied statistical algorithm is described in more detail in the article by Mishra and Macgregor (32). Briefly, we grouped SNPs within the miRNA loci ± 20 kb flanking sequence together and calculated a set-based test statistic as the sum of the χ^2^ one degree of freedom association *P*-values within the miRNA. The observed test statistic was compared with simulated test statistics from the multivariate normal distribution with correlation equal to the corresponding LD structure as derived from the 1,000 Genomes phase 3 European population genotypes (35, 36). We calculated an empirical miRNA-based *P*-value as the proportion of simulated test statistics above the observed test statistic. For the purposes of the present study, we used the top-0.1-test option which summarizes the 10% most significant SNPs for each miRNA.

Using the two summary statistics, miRNA-based *P*-values were calculated for all miRNAs. The calculated miRNA-based *P*-values were corrected for multiple testing according to Benjamini-Hochberg.

### Enrichment tests

To test whether nominally significant SNPs were enriched within miRNAs and their flanking regions, we conducted the Fisher's Exact Test for each summary statistic separately. Additionally, we tested whether the number of cis-miR-eQTL SNPs identified by Huan et al. (37) with a *p*-value of < 0.05 was higher than expected using the Fisher's Exact Test.

## Results

Of the nine tested BD-associated miRNAs, *miR-499a* showed nominally significant *P*-values in both datasets (dichotomous and continuous treatment response, Table 1). Of the remaining 1,692 miRNAs tested for the genome-wide miRNA analysis, 71 miRNAs showed nominally significant associations with the dichotomous and 106 with the continuous treatment response. Fifteen miRNAs revealed nominal significance with both phenotypes. *miR-633* showed the strongest association with the continuous phenotype (*p* = 9.80E-04). Regarding the dichotomous phenotype, *miR-607* showed the strongest association (*p* = 5.79E-04). No association between miRNAs and treatment response to lithium in BD in either of the tested conditions withstood multiple testing correction (Tables 1, 2).

**Table 1:** Results of the window-based tests for the nine BD-associated miRNAs.

**miRNA [position]**	**n SNPs**	**p miRNA**	**p_corr_ miRNA**	**Top SNP [position]**	**p Top SNP**
**A) Dichotomous Treatment Response Measure**					
*miR-499a* [chr20:34990376-34990497]	87	1.71E-02	9.31E-01	rs117616040 [chr20:34977725]	2.27E-03
*miR-135a-1* [chr3:52294219-52294308]	41	9.29E-02	9.31E-01	rs699465 [chr3:52276426]	1.98E-02
*let-7g* [chr3:52268278-52268361]	44	1.04E-01	9.31E-01	rs699465 [chr3:52276426]	1.98E-02
*miR-644a* [chr20:34466325-34466418]	35	2.03E-01	9.37E-01	rs7266300 [chr20:34449603]	9.76E-03
*miR-708* [chr11:79402022-79402109]	130	3.51E-01	9.50E-01	rs12275848 [chr11:79416285]	7.89E-02
*miR-1908* [chr11:61815161-61815240]	39	4.33E-01	9.50E-01	rs61897792 [chr11:61819414]	3.46E-02
*miR-640* [chr19:19435063-19435158]	44	6.08E-01	9.55E-01	rs79954596 [chr19:19437834]	1.74E-01
*miR-611* [chr11:61792495-61792561]	33	6.36E-01	9.55E-01	rs17762402 [chr11:61785729]	4.94E-02
*miR-581* [chr5:53951504-53951599]	55	6.41E-01	9.55E-01	rs72644081 [chr5:53949850]	5.45E-02
**B) Continuous Treatment Response Measure**					
*miR-499a* [chr20:34990376-34990497]	87	3.18E-02	7.52E-01	rs117616040 [chr20:34977725]	3.73E-03
*miR-708* [chr11:79402022-79402109]	130	7.37E-02	8.02E-01	rs1355423 [chr11:79420556]	2.05E-02
*miR-611* [chr11:61792495-61792561]	33	9.79E-02	8.22E-01	rs174532 [chr11:61781402]	3.30E-03
*miR-644a* [chr20:34466325-34466418]	35	1.95E-01	8.50E-01	rs7266300 [chr20:34449603]	6.47E-02
*miR-1908* [chr11:61815161-61815240]	39	2.45E-01	8.64E-01	rs968567 [chr11:61828092]	3.20E-02
*miR-640* [chr19:19435063-19435158]	45	2.69E-01	9.52E-01	rs79954596 [chr19:19437834]	1.15E-02
*let-7g* [chr3:52268278-52268361]	44	6.40E-01	9.81E-01	rs58315325 [chr3:52261812]	2.80E-02
*miR-135a-1* [chr3:52294219-52294308]	41	7.81E-01	9.86E-01	rs34135146 [chr3:52279416]	6.28E-02
*miR-581* [chr5:53951504-53951599]	55	9.22E-01	7.52E-01	rs697112 [chr5:53964849]	1.61E-01

*microRNAs (miRNAs) are sorted according to their miRNA-based P-value. Genome build used is GRCh38 (hg38). Abbreviations: miRNA, microRNA; position, genomic position; nSNPs, number of investigated SNPs; p miRNA, miRNA-based P-value; p_corr_ miRNA, Benjamini-Hochberg corrected miRNA-based P-value; Top SNP, top single-nucleotide polymorphism within gene; p Top SNP, P-value of the Top SNP within gene*.

**Table 2:** Results of the window-based tests for the top five genome-wide miRNAs.

**miRNA [position]**	**n SNPs**	**p miRNA**	**p_corr_ miRNA**	**Top SNP [position]**	**p Top SNP**
**A) Dichotomous Treatment Response Measure**					
*miR-607* [chr10:96828669-96828764]	48	5.79E-04	9.31E-01	rs111682442 [chr10:96823685]	2.73E-04
*miR-8085* [chr19:44758657-44758721]	44	2.54E-03	9.31E-01	rs7249244 [chr19:44742441]	1.11E-04
*miR-1296* [chr10:63372957-63373048]	62	4.13E-03	9.31E-01	rs10995527 [chr10:63387659]	5.47E-03
*miR-4535* [chr22:48780295-48780353]	160	4.62E-03	9.31E-01	rs131016 [chr22:48775746]	1.24E-03
*miR-451b* [chr17:28861371-28861438]	44	5.11E-03	9.31E-01	rs34901720 [chr17:28852500]	2.17E-03
**B) Continuous Treatment Response Measure**					
*miR-633* [chr17:62944215-62944312]	29	9.80E-04	7.52E-01	rs1588368 [chr17:62938848]	1.21E-04
*miR-6516* [chr17:77089417-77089497]	183	1.97E-03	8.02E-01	rs2411054 [chr17:77074245]	2.68E-05
*miR-218-1* [chr4:20528275-20528384]	105	2.13E-03	8.22E-01	rs540146 [chr4:20544433]	6.08E-04
*miR-7704* [chr2:176188843-176188901]	68	2.20E-03	8.50E-01	rs7589870 [chr2:176208720]	1.26E-03
*miR-548e* [chr10:110988926-110989013]	54	2.48E-03	8.64E-01	rs1327551 [chr10:111008438]	1.09E-03

*microRNAs (miRNAs) are sorted according to their miRNA-based P-value. Genome build used is GRCh38 (hg38). Abbreviations: miRNA, microRNA; position, genomic position; nSNPs, number of investigated SNPs; p miRNA, miRNA-based P-value; p_corr_ miRNA, Benjamini-Hochberg corrected miRNA-based P-value; Top SNP, top single-nucleotide polymorphism within gene; p Top SNP, P-value of the Top SNP within gene*.

The number of nominally significant SNPs in both of our GWAS of lithium response located at miRNA loci (*n* = 6,321 and *n* = 5,742 for continuous and dichotomous measurement, respectively) was not significantly higher than expected (*p* = 9.96E-01 and *p* = 1 for continuous and dichotomous measurement, respectively, Fisher's Exact Test).

The number of cis-miR-eQTL SNPs [identified by Huan et al. (37) in our summary statistics (*n* = 341 and *n* = 318 for continuous and dichotomous measurement, respectively) were significantly higher than expected (*p* = 3.31E-05 and *p* = 6.23E-03 for continuous and dichotomous measurement, respectively, Fisher's Exact Test).

## Discussion

The current study investigated whether common variants at BD-associated miRNA, or any other miRNA loci, contribute to the differences in lithium response in BD patients.

*miR-499a* showed a nominally significant association with lithium response in the candidate approach. Although the association did not withstand correction for multiple testing, this result provides some evidence that *miR-499a* might be involved in both development and treatment of BD. A previous study has shown an upregulation of this miRNA in the prefrontal cortex of patients with depression (38). In another study, *miR-499a* was differentially expressed in the postmortem brains of BD patients compared with controls (39). Furthermore, a recent study by Banach et al. (40) reported lower expression levels of *miR-499* in the peripheral blood of BD patients during depressive episodes in comparison to remission, suggesting *miR-499* as a potential new biomarker of illness state in BD.

Overall, the results of our candidate approach do not suggest that individual BD-associated miRNAs might have a strong influence on differential responses to lithium treatment in BD as no association withstood multiple testing correction. On the one hand, this might at least in part reflect that the power to detect associations between common variants and lithium response was limited in the present study, even though the ConLiGen GWAS comprised several thousand individuals (41). On the other hand, it might also indicate that the genetic factors that contribute to BD etiology are different from those contributing to treatment response or illness course. That there are such effects in multifactorial diseases is supported by a study in ulcerative colitis in which no SNPs from 163 inflammatory bowel disease susceptibility loci (42) were found to be associated with the disease course (43).

In our systematic, genome-wide analysis of miRNAs, 106 miRNAs revealed nominally significant associations with the continuous and 71 with the dichotomous lithium treatment response.

The intergenic *miR-633* located on chromosome 17 showed the strongest association with the continuous phenotype (*p* = 9.80E-04). To date, few published studies have investigated the function of *miR-633*. Interestingly, one study reported that *miR-633* was differentially regulated in the cerebrospinal fluid of patients with multiple sclerosis compared to patients with other neurologic diseases. In addition, *miR-633* differentiated relapsing-remitting from secondary progressive multiple sclerosis courses suggesting this miRNA as a potential biomarker for disease course in multiple sclerosis (44).

*miR-607*, an intergenic miRNA located upstream of the ligand dependent nuclear receptor corepressor (*LCOR*) gene on chromosome 10, displayed the strongest association with the dichotomous treatment response measure (*p* = 5.79E-04). The function of this miRNA has been poorly characterized so far, so that we cannot currently speculate about possible disease- and treatment-relevant biological processes. Further research is needed to elucidate the potential role of *miR*-*607* in health or disease.

No association between miRNAs and BD treatment response to lithium in either of the tested conditions withstood multiple testing correction. In addition, we did not observe a significant enrichment for SNPs at all microRNA loci in the present study. Given the limited power of our study, future investigation of miRNAs in larger GWAS samples of BD and lithium response is warranted as better understanding of genetic factors contributing to disease etiology and treatment response might enable the individualization of treatment as well as the identification of novel therapeutic targets (45).

In the present study, we investigated all currently known miRNAs regardless of their tissue or developmental expression patterns. Approximately 70% of ncRNAs are thought to be brain expressed (23) and are dynamically regulated during development and over the lifespan. While the exact mechanisms by which lithium exerts its therapeutic effects remain unclear, pharmacokinetics and pharmacodynamics highlight the importance of specific tissues (e.g., brain and kidney) in treatment responsiveness (46, 47). Therefore, an analysis including miRNAs expressed specifically in these tissues would seem to be a rational follow-up step to reduce the multiple testing burden and to narrow-down the miRNAs to those that *a priori* may have a greater chance to be involved in lithium response. Unfortunately, a systematic enrichment analyses for miRNAs in particular tissues would be premature, since there are currently no comprehensive expression databases derived from normal tissue covering all known miRNAs investigated in the present study. Data on miRNA expression at various developmental stages is also still limited, as non-polyadenylated transcripts are typically not captured with standard library preparation for RNA sequencing. Furthermore, some miRNAs may only be expressed during early developmental stages but can still have an influence on lithium response later on in life, particularly if expression is induced by pharmaceutical treatment. Nevertheless, these aspects remain important and should be considered in future analyses as soon as more comprehensive data on miRNA expression become available.

Using the present approach, we were not able to investigate SNPs with trans-expression quantitative trait loci (eQTL) effects on miRNAs. Previous studies suggest that a substantial proportion of the identified miR-eQTLs are trans-eQTLs (48). Therefore, future investigations into the molecular interactions underlying the association between miRNA trans-eQTLs and treatment response to lithium in BD are also warranted. Huan et al. (37) conducted a genome-wide miR-eQTL mapping study and found consistent evidence for 5,269 cis-miR-eQTLs for 76 mature microRNAs. The significant enrichment for cis-miR-eQTL SNPs found in our summary statistics provides some evidence for the importance of cis-miR-eQTLs in lithium response, although we were not able to identify cis-miR-eQTL SNPs in our top findings since those miRNAs were not among the 76 mature microRNAs reported by Huan et al. (37).

Moreover, miRNAs only represent one class of non-coding RNAs. In the ConLiGen GWAS a genome-wide significant locus containing two lncRNAs was identified (14). Further analyses on the contribution of lncRNAs to lithium response are therefore warranted. This was beyond the scope of the present analysis as the current understanding of the predicted structure of lncRNA molecules and their biological functions remains limited (49).

In conclusion, our analyses do not provide strong evidence that miRNAs are involved in individual response to lithium treatment in BD, as no association between miRNAs and lithium response withstood multiple testing correction. Our data should still be interesting for follow-up of independent studies, particularly when sufficient data is available to accurately define the tissue and temporal expression profile of all human miRNAs, which would allow a more targeted analysis of brain-expressed miRNAs, thereby reducing the search space to miRNAs with relevant expression profiles. We did not find any strong effect that could be useful in terms of a personalized treatment for individual patients. This does not exclude a possible (small) effect of miRNAs on lithium response, and further independent and even larger studies should be envisaged to clarify this question. In parallel, the investigation of other biological mechanisms possibly contributing to lithium treatment response may provide insights for individualizing future pharmacotherapy in BD.

## Author contributions

CR, AF, MN, and SC contributed to the conception and design of the study. AF, LH, UH, FD, MA, KA, NA, RA, BA, LB, AB, SB, AKB, JB, ABi, CM-C, PC, G-BC, H-CC, CC, SRC, FC, DC, CCr, PMC, AD, BE, PF, LF, JF, SG, JG, FG, PG, OG, RH, JHa, SH, SJ, EJ, J-PK, LK, SK-S, SK, BK, IK, NL, GL, ML, CL, MLe, SL, CAL, GM, MM, LM, MMa, MJM, SM, MMi, FM, PM, CN, UÖ, NO, RP, AP, DR-E, GR, PRS, KOS, BS, FS, GS, TS, PDS, KS, CS, CMS, JS, AS, TSt, PS, ST, AT, GT, JV, SW, AW, LY, PZ, JP, JD, MB, ER, TN, J-MA, MMaj, BB, PM, EV, MF, JR, P-HK, TK, MG-S, AR, MD, FB, MS, NW, JK, MAl, FM, TGS, MR, and MN recruited the patients and contributed genotype data. CR and JH performed the statistical analysis. CR, AF, MR, MN, and SC prepared the manuscript, with feedback from the other authors. All authors contributed to manuscript revision, read and approved the submitted version.

### Conflict of interest statement

The authors declare that the research was conducted in the absence of any commercial or financial relationships that could be construed as a potential conflict of interest.
